# Correlation of Culture Positivity, PCR Positivity, and Burden of *Borrelia burgdorferi* Sensu Lato in Skin Samples of Erythema Migrans Patients with Clinical Findings

**DOI:** 10.1371/journal.pone.0136600

**Published:** 2015-09-09

**Authors:** Daša Stupica, Lara Lusa, Vera Maraspin, Petra Bogovič, Darja Vidmar, Maria O’Rourke, Andreas Traweger, Ian Livey, Franc Strle

**Affiliations:** 1 Department of Infectious Diseases, University Medical Center Ljubljana, Ljubljana, Slovenia; 2 Institute for Biostatistics and Medical Informatics, Faculty of Medicine, Ljubljana, Slovenia; 3 Vaccine R & D, Baxter Innovations, Orth an der Donau, A-2304, Austria; University of Kentucky College of Medicine, UNITED STATES

## Abstract

**Background:**

Limited data are available regarding the relationship of *Borrelia* burden in skin of patients with erythema migrans (EM) and the disease course and post-treatment outcome.

**Methods:**

We studied 121 adult patients with EM in whom skin biopsy specimens were cultured and analyzed by quantitative PCR for the presence of *Borreliae*. Evaluation of clinical and microbiological findings were conducted at the baseline visit, and 14 days, 2, 6, and 12 months after treatment with either amoxicillin or cefuroxime axetil.

**Results:**

In 94/121 (77.7%) patients *Borrelia* was detected in skin samples by PCR testing and 65/118 (55.1%) patients had positive skin culture result (96.8% *B*. *afzelii*, 3.2% *B*. *garinii*). *Borrelia* culture and PCR results correlated significantly with the presence of central clearing and EM size, while *Borrelia* burden correlated significantly with central clearing, EM size, and presence of newly developed or worsened symptoms since EM onset, with no other known medical explanation (new or increased symptoms, NOIS). In addition, the logistic regression model for repeated measurements adjusted for time from inclusion, indicated higher *Borrelia* burden was a risk factor for incomplete response (defined as NOIS and/or persistence of EM beyond 14 days and/or occurrence of new objective signs of Lyme borreliosis). The estimated association between PCR positivity and unfavorable outcome was large but not statistically significant, while no corresponding relationship was observed for culture positivity.

**Conclusions:**

Higher *Borrelia* burden in EM skin samples was associated with more frequent central clearing and larger EM lesions at presentation, and with a higher chance of incomplete response.

## Introduction

Erythema migrans (EM) is the clinical hallmark of early Lyme borreliosis (LB), the most common tick-borne human disease in the Northern Hemisphere, caused by *Borrelia burgdorferi* s.l. [1–3]. Although antibiotic therapy is highly effective in resolving this manifestation, a proportion of patients (from zero to 40.8% in the USA and from zero to 23.4% in Europe [4] complain of persistent subjective symptoms lasting for more than 6 months after treatment, termed post-LB symptoms. The potential trigger mechanisms of these symptoms remain unclear and may be multifactorial. In our previous study we suggested that *Borrelia* skin culture positivity may predict a less favorable treatment outcome in EM patients [5]. Skin culture positivity was found to be significantly associated with larger numbers of spirochetes in EM skin lesion specimens [6–8], suggesting that borrelial burden in the skin influences the course and post-treatment outcome of the disease. To further investigate the pre-treatment course and the post-treatment outcome in EM patients, we compared demographic, epidemiological, clinical, and microbiological findings according to the quantity of *B*. *burgdorferi* sensu lato as detected by quantitative PCR of EM skin samples taken before antibiotic treatment. We hypothesized that a higher borrelial burden in the skin of EM patients might influence the pre-treatment course and post-treatment outcome of the disease.

## Methods

### Setting and Patients

Patients were evaluated from June through December 2010 at the LB Outpatient’s Clinic, University Medical Center Ljubljana, Slovenia. Patients were enrolled in the study if they were ≥15 years old, with typical solitary EM defined according to the criteria reported by Stanek et al [9]. Patients were excluded if they did not consent to the skin biopsy; had EM localized on head, neck or breasts; were pregnant or lactating; had received an antibiotic with known anti-borrelial activity within 10 days; had multiple EM lesions; had an extracutaneous manifestation of LB; or had an intercurrent episode of LB during follow-up. Patients received cefuroxime axetil 500 mg twice daily, or amoxicillin 500 mg three times daily for 15 days. The antibiotic treatment option was varied weekly during study entry.

The study was approved by the Medical Ethics Committee of the Ministry of Health of the Republic of Slovenia, No 127/06/10. All patients gave written consent for participation. For the two patients aged < 18 years, we additionally obtained their parents’ written consent.

Results on quantitative detection *of Borrelia burgdorferi* sensu lato in EM skin lesions of patients included in the present study have been reported in detail previously, together with some of the pretreatment patients' characteristics [8].

### Evaluation of Patients

At all study visits (baseline, 14 days, 2, 6, and 12 months post-enrollment), participants underwent a physical exam and were asked an open question about health-related symptoms. Systemic symptoms that newly developed or had worsened since the onset of EM, but had no other known medical explanation were regarded as new or increased symptoms (NOIS). At day 14, patients were asked about medication compliance and adverse events.

Complete response was defined as return to pre-LB health status, while incomplete response was delineated as the presence of NOIS (partial response) and/or occurrence of new objective signs of LB, and/or persistence of *B*. *burgdorferi* sensu lato in skin as detected by culture at the site of the previous EM, and/or persistence of EM at 2 months or beyond post-treatment (failure). Persistence of EM means that EM could still be noticed on day light and at room temperature.

### Microbiological analysis

At baseline serum IgM and IgG antibodies to C6 peptide derived from the VlsE protein of *B*. *burgdorferi* sensu lato were determined using the C6 Lyme ELISA kit (Immunetics).

At baseline, paired, 3 mm, punch biopsies were obtained from the expanding edge of EM. One of the skin specimens was placed directly in a tube containing 6 ml BSK-B medium for culture, the second was placed in 70% ethanol for quantitative PCR analysis. Skin biopsy was repeated 2–3 months after the first biopsy in patients in whom the first skin biopsy specimen was culture positive for *B*. *burgdorferi* sensu lato. Cultures were examined weekly for the presence of spirochetes by dark-field microscopy and were interpreted as negative if no growth was established up until 12 weeks [8]. Blood cultures for the presence of *Borrelia* were performed as previously described [10].

DNA was extracted from skin biopsy specimens using a QIAamp DNA Mini kit (Qiagen). A quantitative real-time TaqMan PCR assay amplifying and targeting a conserved 139bp fragment of the gene encoding the *Borrelia* 16S rRNA was used to detect borrelial nucleic acids in total DNA extracted from skin specimens. Real-time PCR assays yielding a Cq value of less than 40 were scored as positive. The number of targets per biopsy reflects the total spirochetal load per biopsy sample, however to normalize the data accounting for differences in biopsy sizes and differences in human skin at the site biopsied, the spirochete load was also expressed per 10,000 human genome equivalents [8]. Isolates were typed according to their *OspA* sequences as outlined previously [11].

### Statistical analysis

Data were summarized as medians with interquartile range (IQR) for numerical variables and as frequencies and percentages for categorical variables. The association between the test results (PCR, culture or number of *Borrelia* 16S rRNA targets) with the other variables was assessed using the chi-squared test with Yates' continuity correction, Mann-Whitney test or Spearman’s correlation; to control for false positives, the *P* values were adjusted using a multivariate permutation procedure [12].

To assess the association of the test results with incomplete response to treatment, we estimated three separate logistic regression models with incomplete response as the dependent variable, and skin culture positivity/PCR positivity/borrelial burden as a covariate. To take into account the multiple measurements repeated in each patient, the analyses were adjusted for a subject variable as a random effect, and for follow-up time (used as a categorical variable).

The shape of the association between EM size and the probability of positive test results was estimated using a logistic regression model, modelling the EM size as a restricted cubic spline; a similar (linear regression) model was used to model the number of *Borrelia* 16S rRNA targets and results were displayed graphically.

All the statistical analyses were performed using the R statistical language [13].

## Results

One hundred twenty one patients with solitary EM, 58.7% of whom were male, with a median age 54 (IQR 43–61) years, were enrolled in the study. Sixty one patients received cefuroxime axetil and 60 were treated with amoxicillin.

Paired skin samples were obtained from all the patients. Three of 121 cultures became contaminated and could not be analyzed further; of the 118 analyzable biopsies 65 (55.1%) were culture-positive. Of 62 identified *Borrelia* species, 60 (96.8%) were *B*. *afzelii* and 2 (3.2%) were *B*. *garinii*. Of the 121 biopsies, 94 (77.7%) were *Borrelia*-positive in PCR assay. All culture-positive biopsies were also positive by PCR. In three patients (2.5%), *Borrelia* was cultured from blood; skin biopsies from these patients were also positive by culture and PCR.

As reported previously [8] the number of *Borrelia* 16S rRNA targets detected by quantitative PCR for PCR positive patients varied from 12 to 17,458 target copies (median 905.5, IQR 188 to 2,415) and from 0.2 to 321 target copies per 10,000 human genome equivalents (median 15, IQR 5 to 39.75). In the present study the bacterial load was expressed in relation to human genomic DNA to compensate for variations arising from the DNA extraction procedure and/or the size and quality of the biopsy sample.

The number of *Borrelia* 16S rRNA targets detected in culture positive specimens (median 22, IQR 6 to 53) was significantly higher than in culture negative specimens (median 6, IQR 2 to 31.5; P<0.001).

In 81 of 118 (68.6%) patients discernible levels of antibody to C6 (C6 ELISA) were detected at presentation Serum samples from 81 of 118 (68.6%) patients tested positive for *Borrelia* antibodies (C6 ELISA) at enrollment (Table 1). Patients with positive serological results more often had positive skin culture and positive PCR results and had a higher borrelial burden than serologically negative patients. The association between serology and PCR positivity (P = 0.01) and serology and borrelial burden (P = 0.01) were statistically significant.

**Table 1:** Association of selected pretreatment characteristics with *Borrelia* skin culture positivity and with *Borrelia* skin PCR positivity.

Characteristic	Culture result 65/118^a^ (55.1%)				PCR result 94/121^b^ (77.7%)			
	Positive (N = 65)	Negative (N = 53)	P value^c^	P adj^d^	Positive (N = 94)	Negative (N = 27)	P value^c^	P adj^d^
Male sex	30 (46%)	18 (34%)	0.25	0.92	43 (46%)	7 (26%)	0.10	0.57
Comorbidities^e^	29 (45%)	26 (49%)	0.77	>0.99	43 (46%)	12 (46%)	1.00	>0.99
Immunocompromised^f^	2 (3%)	4 (8%)	0.50	>0.99	4 (4%)	2 (7%)	0.87	>0.99
Tick bite^g^	29 (45%)	23 (43%)	1.00	>0.99	41 (44%)	13 (48%)	0.84	>0.99
Days since EM first observed	7 (5–10)	7 (4.3–13)	0.83	>0.99	7 (5–10)	7 (4–14)	0.89	>0.99
EM with central clearing	33 (51%)	18 (34%)	0.10	0.56	47 (50%)	6 (22%)	**0.02**	0.13
Diameter of EM, cm	14 (10–21)	14 (9–20)	0.47	>0.99	14 (10–20)	10 (5.5–17)	**0.01**	**0.04**
Systemic symptoms^h^	17 (26%)	11 (21%)	0.64	>0.99	26 (28%)	3 (11%)	0.13	0.64
Fatigue	9 (14%)	6 (11%)			14 (15%)	2 (7%)		
Headache	4 (6%)	7 (13%)			10 (11%)	1 (4%)		
Myalgia	2 (3%)	4 (8%)			5 (5%)	1 (4%)		
Arthralgia	4 (6%)	1 (2%)			5 (5%)	0 (0%)		
Number of symptoms^i^	1 (1–2)	2 (1–4)	0.15	>0.99	1 (1–2)	2 (1.5–3)	0.40	0.19
Symptoms at EM site	23 (35%)	23 (43%)	0.49	>0.99	35 (37%)	13 (48%)	0.42	>0.99
Seropositive^j^	48 (75%)	32 (62%)	0.17	0.82	71 (76%)	71 (76%)	**<0.01**	**0.01**
Treated with amoxicillin	35 (54%)	24 (45%)	0.46	>0.99	48 (51%)	12 (44%)	0.70	>0.99

Data are median (interquartile range) or number (%) of patients.

Abbreviations: adj, adjusted; EM, erythema migrans.

^a^ Number of *Borrelia*-positive skin culture results out of the 118 analyzable biopsies.

^b^ Number of *Borrelia*-positive PCR results out of 121 biopsies.

^c^ P value for comparison between culture positive vs culture negative and between PCR positive vs PCR negative patients.

^d^ Adjusted P value for multiple comparisons.

^e^ Patients with underlying chronic illness such as arterial hypertension, hyperlipidemia, osteoporosis, diabetes mellitus, thyroid disease, cardiac rhythm abnormality, psychiatric illness, ischemic heart disease, osteoarthritis, asthma.

^f^ 11 patients had active malignancy, one patient had Crohn’s disease.

^g^ Patients with history of a tick bite at the site of the EM skin lesion.

^h^ Patients with EM who reported new or increased systemic symptoms (NOIS). Some patients had more than one NOIS.

^i^ Number of NOIS in patients who reported NOIS.

^j^ Immunoglobulin M and/or immunoglobulin G to *B*. *burgdorferi* sensu lato positive at enrollment. Serological testing was done for 64 culture-positive patients and for 52 culture-negative patients, for 93 PCR-positive, and for 25 PCR-negative patients.

### Pre-treatment characteristics according to culture positivity, PCR positivity and *Borrelia* burden

**Fig 1:**
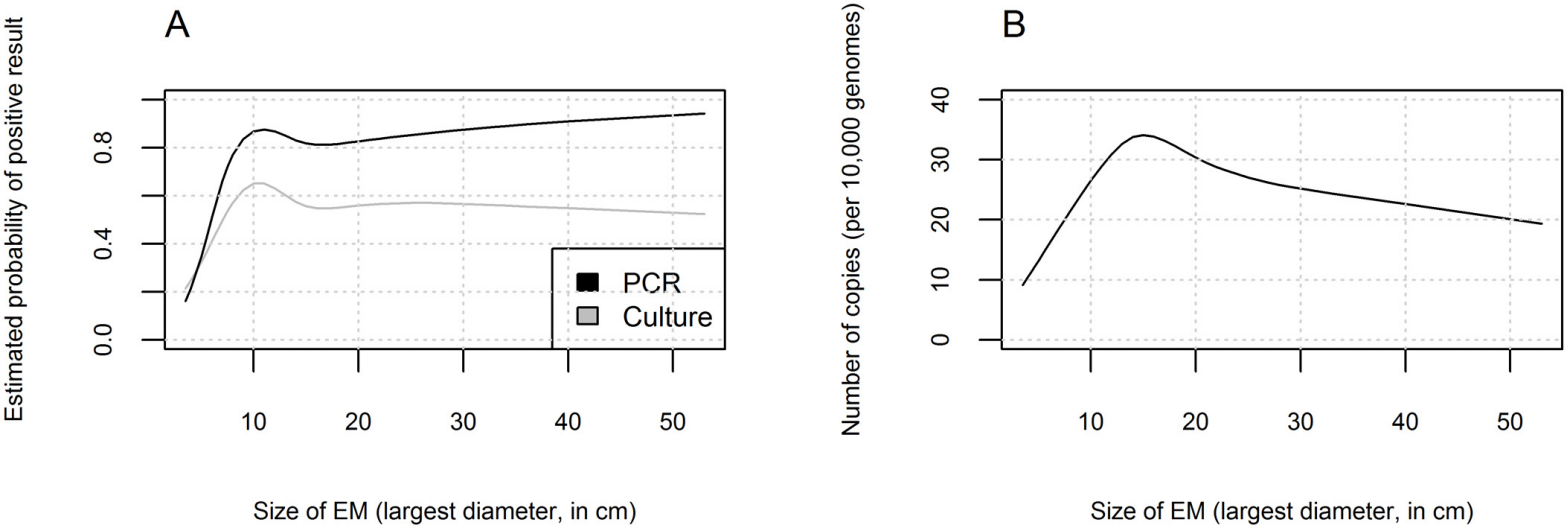
A. Estimated associations between erythema migrans (EM) size and culture positivity, and between EM size and PCR positivity estimated with logistic regression model. B. Estimated associations between EM size and *Borrelia* burden (spirochete copies per 10,000 genome equivalents), estimated with a linear regression model. Restricted cubic splines were used to flexibly model the association between EM size and the outcomes.

**Fig 2:**
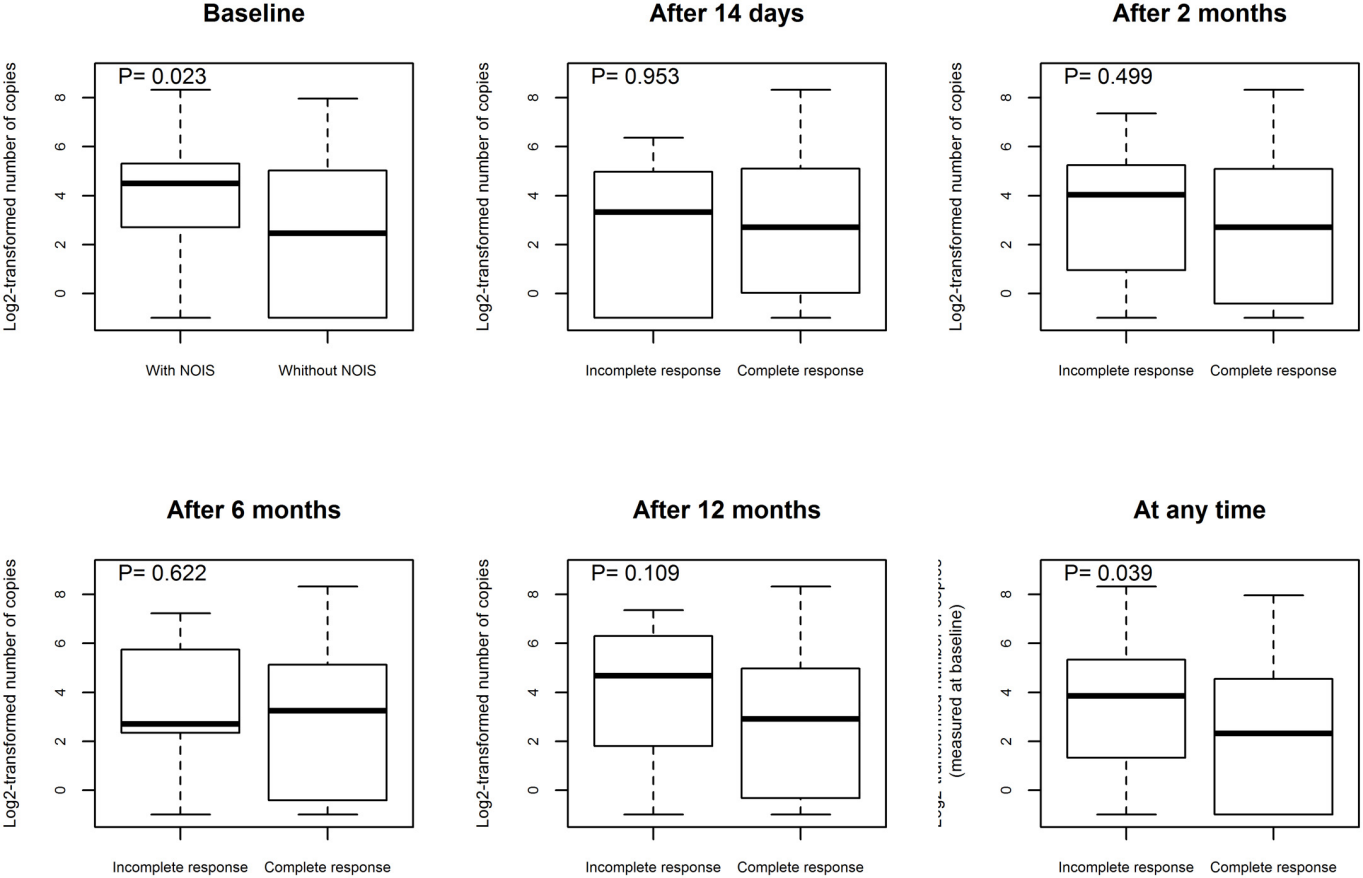
Associations between *Borrelia* burden at presentation and presence of new or increased symptoms (NOIS) at different time points using the Mann-Whitney test.

Patients characteristics at enrollment with regard to skin culture positivity and PCR results are outlined in Table 1. The EM lesions of patients with positive culture or PCR results exhibited central clearing more often and were typically larger in size; these types of EM also exhibited larger *Borrelia* burden. The association between these EM characteristics and the test results was stronger when the PCR, rather than the culture result, was used (Table 1). Interestingly, the estimated shapes of associations between EM size and culture positivity, PCR positivity, or *Borrelia* burden were similar (Fig 1). The estimated probability of having a positive culture or PCR result increased sharply with the size of EM for patients with EM smaller than 10 cm and remained stable for EM skin lesions ≥ 10 cm (Fig 1). Similarly, the estimated average *Borrelia* burden increased with the size of EM for patients with EM skin lesions ≤ 15 cm (Fig 1). The proportion of patients reporting NOIS at enrollment was higher in culture or PCR-positive vs. culture or PCR-negative patients, although the differences were not statistically significant (Table 1), however patients with NOIS at enrollment had a statistically significantly higher *Borrelia* burden (Fig 2). Among NOIS, fatigue was the most commonly reported systemic symptom, followed by headache, myalgia, and arthralgia. (Table 1).

### Treatment outcome

All patients were compliant with taking the study drug assigned. No adverse event resulted in discontinuation of treatment.

In 61 of the 65 culture-positive patients re-biopsy was performed. All of the re-biopsies were culture-negative. Sixty re-biopsies were also PCR-negative while one re-biopsy was PCR-positive. The patient with PCR-positive re-biopsy did not have systemic symptoms at presentation. His skin lesion resolved in three days after starting antibiotics and he remained asymptomatic up till the 12-month visit. The median duration of EM skin lesion after starting antibiotic treatment did not differ significantly with regard to *Borrelia* burden. Failure was documented in two patients, both of whom were PCR and culture-positive, and both received amoxicillin. One of these patients developed meningitis by the 14-day visit and was given ceftriaxone and recovered uneventfully. The other patient was retreated with azithromycin because of faint residua of EM skin lesion persisting at the 6-month follow-up visit, which could still be detected at the 12-month visit, but no biopsy was performed for histopathology to assess whether the lesion represented active inflammatory disease. It is questionable whether this case truly represented a treatment failure because pale residua at the location of previous EM skin lesion may have represented post-inflammatory hyperpigmentation.

The large majority of patients (71% of PCR-positive patients and 81.5% of PCR-negative patients) had complete response from 2 months onward returning to pre-LB health status.

**Fig 3:**
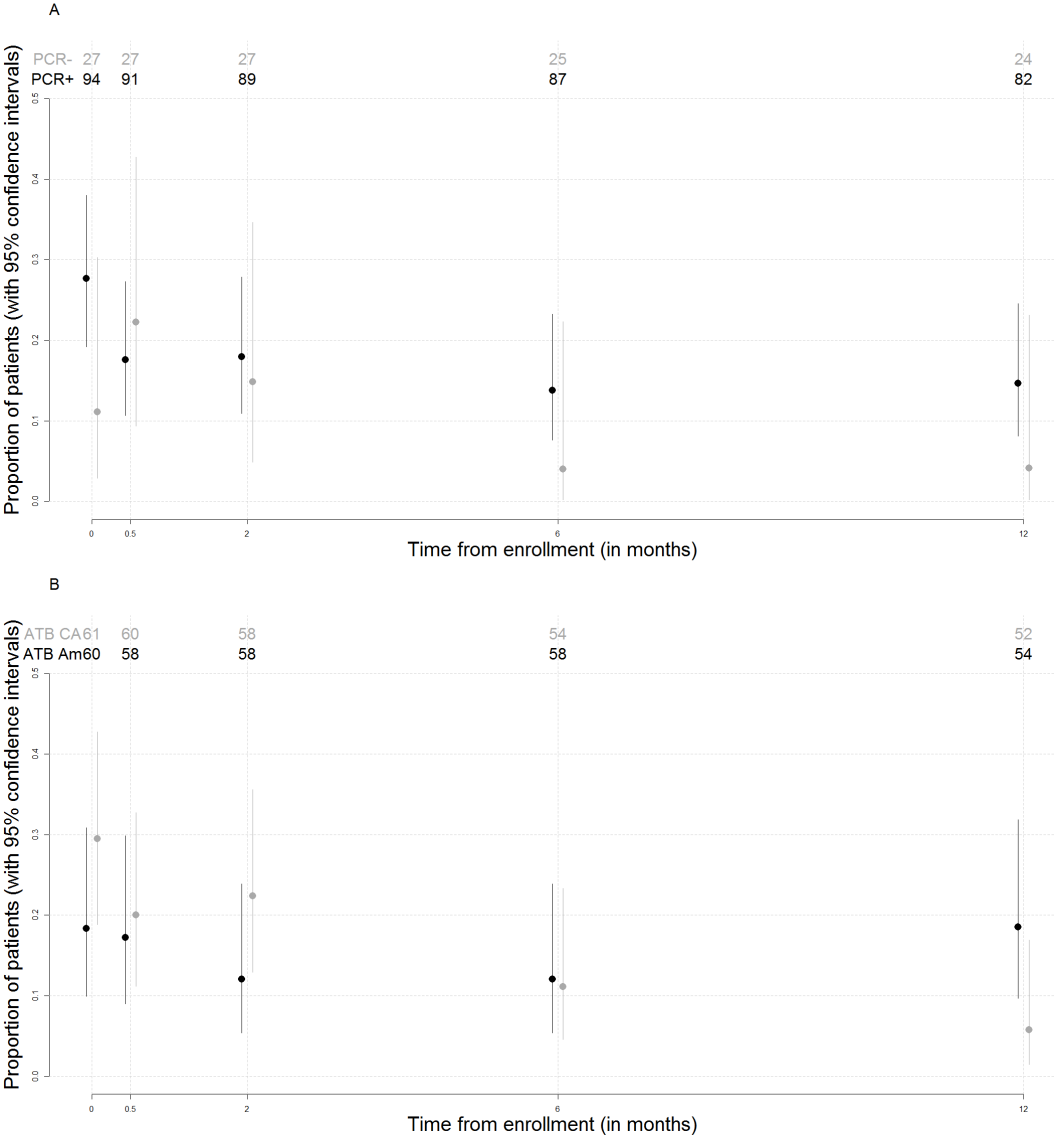
A. Proportion of patients with new or increased symptoms (NOIS) at baseline or incomplete response at follow-up visits in relation to the PCR result of EM lesion at presentation Associations between time from enrollment and proportion of patients reporting new or increased symptoms (NOIS) according to PCR positivity. B. Proportion of patients with new or increased symptoms (NOIS) at baseline or incomplete response at follow-up visits in relation to the antibiotic used for treatment.

Patients in whom EM biopsies tested positive by PCR reported more NOIS at baseline and incomplete responses at 2, 6 and 12 months after enrollment (Fig 3). The logistic regression model for repeated measurements of incomplete response, with PCR positivity as explanatory variable, adjusted for time from enrollment (Table 2, Model B), indicated that the probability of incomplete response significantly decreased with time from enrollment (P = 0.05), most markedly between baseline and the first follow-up time point (OR for incomplete response at 14-day visit versus baseline equal to 0.69, 95% CI from 0.35 to 1.35, P = 0.27) and between second and sixth month (OR = 0.59, 95% CI: 0.27 to 1.32, P = 0.20). PCR-positive patients had a higher probability of incomplete response (OR = 1.83, 95% 0.84 to 3.95, P = 0.13), but the association with incomplete response was not statistically significant.

**Table 2:** Association between incomplete response and time from enrollment and culture positivity (Model A), PCR positivity (Model B), or *Borrelia* skin burden (log2 transformed, Model C).

	Model A: including culture positivity		Model B: including PCR positivity		Model C: including *Borrelia* burden	
	OR (95% CI)	P value	OR (95% CI)	P value	OR (95% CI)	P value
Intercept	0.23 (0.12–0.43)	<0.01	0.15 (0.07–0.35)	<0.01	0.18 (0.09–0.33)	<0.01
Culture (positive vs. negative)	1.08 (0.58 to 2.03)	0.81	**-**	**-**	**-**	**-**
PCR (positive vs. negative)	**-**	**-**	1.83 (0.84 to 3.95)	0.13	**-**	**-**
Borrelial burden (log 2)	**-**	**-**	**-**	**-**	1.12 (1.00 to 1.26)	**0.05**
Time from diagnosis		**0.05**	**-**	**0.05**	**-**	**0.04**
Time 14d vs baseline	0.72 (0.36 to 1.42)	0.34	0.69 (0.35 to 1.35)	0.27	0.68 (0.35 to 1.34)	0.27
Time 2m vs 14d	0.90 (0.44 to 1.84)	0.77	0.90 (0.44 to 1.84)	0.77	0.89 (0.44 to 1.83)	0.76
Time 6m vs 2m	0.59 (0.26 to 1.32)	0.20	0.59 (0.27 to 1.32)	0.20	0.59 (0.26 to 1.31)	0.20
Time 12m vs 6m	0.96 (0.40 to 2.33)	0.93	1.06 (0.45 to 2.52)	0.89	1.07 (0.45 to 2.54)	0.88

Abbrevations: OR, odds ratio for incomplete response.

A similar logistic regression model was used with *Borrelia* burden as an explanatory variable, which indicated that patients with higher *Borrelia* burden had a higher probability for incomplete response (OR = 1.12, 95% CI: 1.00 to 1.26, P = 0.05, Table 2 Model C). Culture positivity was not associated with incomplete response (OR = 1.08, 95% CI: 0.58 to 2.03, P = 0.81, Table 2 Model A).

The frequency of NOIS decreased with time after treatment, however patients treated with amoxicillin reported more NOIS at the 12-month visit (8/53, 15%, 95% CI: 6.7 to 27.6%) compared to patients treated with cefuroxime (3/52, 5.8%, 95% CI: 1.2 to 15.9%) (Fig 3); the difference was not statistically significant, but its clinical meaningfulness could not be excluded (estimated difference in percentage of patients with NOIS: 9.3%, 95% CI: -4 to 22.8%). Furthermore, when the two treatment failures which occurred in the group of patients treated with amoxicillin were taken into account, the difference in the probability of incomplete response between the two treatment regimens was even more pronounced.

## Discussion

Antibiotic treatment of EM is very successful in terms of faster resolution of the skin lesion and accompanying symptoms, and more importantly it is highly effective in preventing development of other objective manifestations of LB [1]. However, a considerable proportion of patients (up to 40%) complain of subjective symptoms ≥6 months after EM treatment. It is not yet clear whether these subjective symptoms are causally associated with antecedent borrelial infection and if so, what the potential underlying mechanisms are. Several therapeutic trials from USA and Europe have independently shown that in patients who reported post-treatment symptoms a greater frequency of multiple EM or accompanying symptoms such as fatigue, headache, arthralgias or myalgias were reported before treatment [14–20]. However, in other studies the severity of the initial illness did not influence treatment outcome [4, 21–23]. Seropositivity to *Borrelia*, either at presentation or at follow up, has uniformly been found not to be a risk factor for unfavorable treatment outcome [4, 16, 17, 20–24]. Strle et al recently reported positive correlation between high T_H_17-associated immune responses in EM patients and the presence of post-Lyme symptoms, suggesting that patient-inherent parameters may influence treatment outcome [25]. In our previous study we evaluated EM skin culture result as a potential influential factor for disease course and treatment outcome. No significant association was ascertained between *Borrelia* skin culture results and selected pre-treatment characteristics, however a higher proportion of patients with incomplete response to treatment in the culture-positive group compared to the culture-negative group indicated less favorable treatment outcome in culture-positive patients [5].

The present study revealed that *Borrelia* load in culture-positive specimens was significantly greater than that detected in culture-negative specimens. This is consistent with findings of Liveris et al, who reported that the average number of spirochetes in culture positive specimens was more than double than in culture negative specimens [6], and by Li et al who found that recovery of *B*. *burgdorferi* in culture correlated with larger numbers of spirochetes in EM lesions [7]. Published reports also indicate that *Borrelia* burden may contribute to the variability of EM clinical presentation [6–8]. Liveris et al found that larger numbers of spirochetes in EM skin lesions were significantly associated with a shorter duration of EM prior to diagnosis, with smaller skin lesions, and infection with a specific genotype of *B*. *burgdorferi* but not with the number or severity of pretreatment constitutional symptoms [6]. Similarly, Li et al reported a higher density of organisms in smaller EM lesions (5–11 cm) during the first days (1–4 days) of illness [7]. These results are consistent with ours, except that in our study peak *Borrelia* density was observed somewhat later [8] and in larger skin lesions than in the aforementioned studies. This “lag” is probably due to the difference of EM evolution with regard to the infecting *Borrelia* species; *B*. *afzelii* being the predominating agent in our study vs. *B*. *burgdorferi* in the studies of Liveris and Li.

However, as opposed to Liveris [6], in this study we found statistically significant association between *Borrelia* burden and the presence of pre-treatment constitutional symptoms accompanying EM.

Not much is known about the potential influence of *Borrelia* skin burden on treatment outcome in EM patients and the present study is the first to directly test the association between the amount of *Borrelia* present in skin lesions of EM patients and the respective treatment outcome.

Out of 121 patients there were two treatment failures (1.7%). In the first case, amoxicillin failure was evidenced by objective findings i.e., progression to meningitis documented by cerebrospinal fluid pleocytosis. The second case with persisting EM, was considered a failure due to our stringent criteria. Both treatment failures occurred among PCR-positive patients and both had uneventful course after being re-treated with antibiotics. Since treatment failure was a rare event in this and in our previous studies [4, 23], patients reporting NOIS represented the great majority in the group with incomplete response. The probability of having NOIS was significantly associated with *Borrelia* burden at the baseline visit, as well overall during follow-up, suggesting that bacterial load in skin could be at least one of the influential parameters for treatment outcome in EM patients.

Patients did not know their PCR results, but they knew their skin culture results. This might have biased our results, which is a drawback of our study. However the steady decline in proportion of symptomatic patients in the PCR-positive as well as in the PCR-negative group contradicts this assumption (Fig 3).

In conclusion, the higher *Borrelia* burden in skin biopsy specimens was associated with a higher chance for constitutional symptoms accompanying EM, but also negatively influenced the post-treatment outcome. Patients with higher *Borrelia* burden were more likely to have incomplete response, represented predominantly by post-Lyme symptoms, implicating that *Borrelia* burden might be involved in the pathogenesis of post-Lyme symptoms. If valid, our assumptions can only be considered relevant for European LB, caused by *B*. *afzelli*.
